# Engineering of atomic-scale flexoelectricity at grain boundaries

**DOI:** 10.1038/s41467-021-27906-0

**Published:** 2022-01-11

**Authors:** Mei Wu, Xiaowei Zhang, Xiaomei Li, Ke Qu, Yuanwei Sun, Bo Han, Ruixue Zhu, Xiaoyue Gao, Jingmin Zhang, Kaihui Liu, Xuedong Bai, Xin-Zheng Li, Peng Gao

**Affiliations:** 1 International Center for Quantum Materials, School of Physics, Peking University, Beijing, 100871 China; 2 Electron Microscopy Laboratory, School of Physics, Peking University, Beijing, 100871 China; 3 Beijing National Laboratory for Condensed Matter Physics and Institute of Physics, Chinese Academy of Sciences, Beijing, 100190 China; 4 State Key Laboratory for Mesoscopic Physics, School of Physics, Peking University, Beijing, 100871 China; 5 Collaborative Innovation Centre of Quantum Matter, Beijing, 100871 China; 6 Interdisciplinary Institute of Light-Element Quantum Materials and Research Center for Light-Element Advanced Materials, Peking University, Beijing, 100871 China; 7 Frontiers Science Center for Nano-optoelectronics, School of Physics, Peking University, Beijing, 100871 China; 8 Peking University Yangtze Delta Institute of Optoelectronics, Nantong, 226010 Jiangsu China

**Keywords:** Ferroelectrics and multiferroics, Surfaces, interfaces and thin films

## Abstract

Flexoelectricity is a type of ubiquitous and prominent electromechanical coupling, pertaining to the electrical polarization response to mechanical strain gradients that is not restricted by the symmetry of materials. However, large elastic deformation is usually difficult to achieve in most solids, and the strain gradient at minuscule is challenging to control. Here, we exploit the exotic structural inhomogeneity of grain boundary to achieve a huge strain gradient (~1.2 nm^−1^) within 3–4-unit cells, and thus obtain atomic-scale flexoelectric polarization of up to ~38 μC cm^−2^ at a 24° LaAlO_3_ grain boundary. Accompanied by the generation of the nanoscale flexoelectricity, the electronic structures of grain boundaries also become different. Hence, the flexoelectric effect at grain boundaries is essential to understand the electrical activities of oxide ceramics. We further demonstrate that for different materials, altering the misorientation angles of grain boundaries enables tunable strain gradients at the atomic scale. The engineering of grain boundaries thus provides a general and feasible pathway to achieve tunable flexoelectricity.

## Abstract (web summary)

Large strain gradient is crucial for flexoelectricity. Here, the authors reveal the generality and tunability of large strain gradients at grain boundaries in oxides, explaining the possible effects on the electrical activities of ceramics.

## Introduction

Flexoelectricity is an emergent phenomenon that describes electric polarization controlled by inhomogeneous deformation, i.e., strain gradients, with broken inversion symmetry^1,2^. The flexoelectric effect has two basic features. One is its inherent universality, which allows the appearance of net polarization from strain gradients for every dielectric material, breaking the limit of piezoelectricity that merely exists in the piezoelectric materials of 20 noncentrosymmetric groups^1,3^. Second, when a given strain field is imposed, strain gradients and hence flexoelectricity are expected to be more considerable with the reduction of the spatial scale^4,5^. Therefore, the flexoelectric effect is pronounced at the nanoscale^6–9^ and has the potential to mediate local polar switching^10,11^, piezoelectric performance^12,13^, optoelectronic characteristics^14^, magnetic properties^15,16^, etc. In particular, dielectric oxides have a large flexoelectric coefficient and thus hold broad promise for application in future nanosized devices^11,17^. However, bending these brittle ceramic materials to realize appropriate and controllable strain gradients is difficult, hindering the achievement of a high flexoelectric response.

Grain boundaries (GBs), which consist of unique periodic arrangements of structural units, can exhibit different properties that are not present in intrinsic bulk crystals^18–22^. For a well-bonded symmetric GB, the atomic configuration of the GB core is a natural trapezoidal shape with a significantly inhomogeneous strain distribution in space. Such disrupted atomic bonding is usually confined to a few unit cells around the GB core, which is expected to introduce huge strain gradients^23^. In addition, tailoring the trapezoidal shape simply by controlling the orientations of two adjacent grains is predictable and practicable, resulting in an adjustable strain gradient as well as flexoelectric polarization. As a result, GBs may serve as a feasible component to realize remarkable and tunable nanoscale flexoelectricity. However, in practice, validating this speculation is challenging, as GBs are of atomic size and have complex structures, while conventional macroscopic characterizations and measurements only provide collective information about the bulk material and may be influenced by other irrelevant factors, such as surface effects^24,25^.

Here, we verify this speculation and develop a general strategy to generate atomic-scale flexoelectric polarization via GB engineering. For a 24° tilt LaAlO_3_ (LAO) GB, we directly visualize the atomic arrangements by advanced atomically resolved scanning transmission electron microscopy (STEM) and spectroscopy techniques and find a huge strain gradient (~1.2 nm^−1^) and a remarkable flexoelectric effect that induces atomic displacement up to 81.4 ± 10.5 pm within 3–4-unit cells around the GB. In such a confined GB region, a large local polarization (~38 μC cm^−2^, estimated by first-principles density functional theory (DFT) calculations) exists, forming a “head-to-head” polarization configuration. Electron energy loss spectroscopy (EELS) illustrates that the flexoelectric phase at the GB has stronger hybridization of La–O interactions. The presence of nanoscale flexoelectricity accompanied by a change in the electronic structures is expected to play a considerable role in the transport properties of electroceramics. Furthermore, when extrapolated to SrTiO_3_ (STO) GBs, huge strain gradients also exist in the 22.6° GB core, and even larger strain gradients are observed in the 36.8° GB, exhibiting that tunable flexoelectricity can be achieved by altering the misorientation angle of GBs in different materials. The demonstrated generality of atomic-scale flexoelectricity at GBs provides essential knowledge to understand the electrical activity of oxide ceramics. The tunability via GB engineering may open vistas in nanoelectronics and nanoelectromechanical systems.

## Results

### GB engineering design

**Fig. 1:**
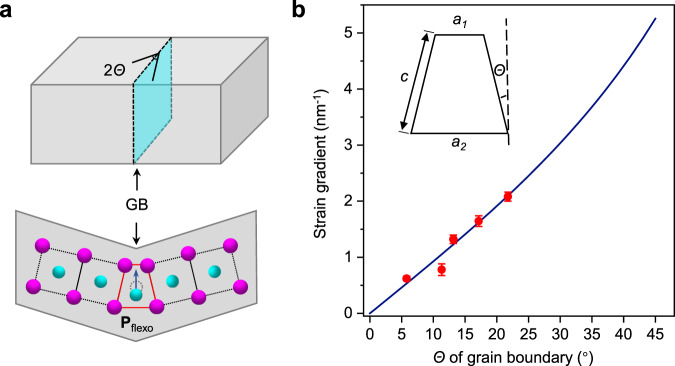
Schematics of the design of a huge strain gradient via GB engineering. **a** Schematic of inhomogeneous deformation of the unit cell in a GB (flexoelectric coefficient *f*_11_ ≠ 0), producing a net dipole moment due to flexoelectricity (**P**_flexo_). **b** Expected distribution of the strain gradient for different GB angles. The red dots represent experimental data from LAO and STO GBs. The error bar is the standard derivation.

Figure 1a shows the distinctive geometry of the GB in which huge strain gradients are achievable. Atoms in a symmetric tilt GB are confined by the perfect bulk matrix, subsequently forming periodic arrangements of trapezoidal structural units with large structural deformation, as highlighted in Fig. 1a. Generally, considerable flexoelectricity can only be obtained from significantly bent crystals^2^. In contrast, such an ideal atomic-sized trapezoid in the GB core is expected to generate a huge strain gradient and introduce a large dipole moment within a single unit cell. Based on the geometric relationship of the trapezoid-shaped unit (see “Methods” for details), we obtain the strain gradients in the GB core for different misorientation angles of the GB (Fig. 1b).

### Atomic-scale GB structure

**Fig. 2:**
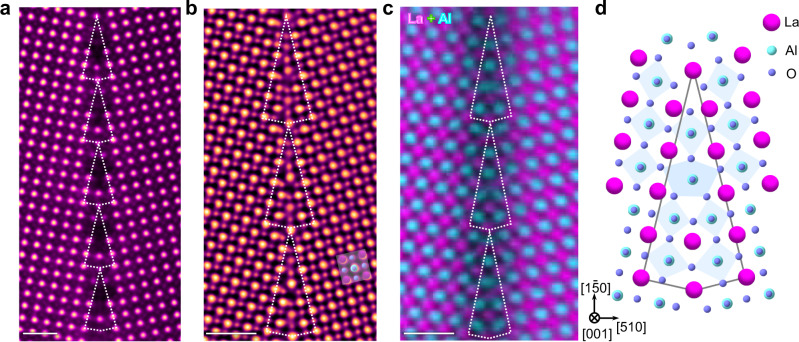
Atomic arrangements of the LAO GB. **a** HAADF image. The periodic structural units are highlighted by the white polygons. Scale bar, 1 nm. **b** iDPC image depicting the oxygen and cationic arrangement. Scale bar, 1 nm. **c** Atomically resolved EDS mapping to determine the cationic configuration in the GB. Magenta: La; cyan: Al. Scale bar, 1 nm. **d** Schematic representation of the complete atomic structure of the LAO GB illustrating structural distortion at the GB. Magenta: La; cyan: Al; purple: O.

Now, we verify the possible presence of flexoelectricity in such a structure by investigating a 24° tilt LAO GB. LAO has no known ferroelectric instability^26^ and is an insulating oxide typically used as a gate dielectric. The LAO bicrystal was fabricated by thermal diffusion bonding^27,28^, the correlative experimental details are presented in Methods. To pinpoint the atomic configurations and chemistries of the inhomogeneity, we employed various advanced STEM techniques. Figure 2a depicts the high-angle annular dark-field (HAADF) image (colored for clarity) viewed along the [001] direction, where structural units are periodically distributed along the [1$\bar{5}$0] projection highlighted by the dotted polygons. The brighter atom columns correspond to La columns, and the other atom type is Al, while the O columns are invisible due to the Z-contrast sensitivity of HAADF imaging. We, therefore, acquired an integrated differential phase contrast (iDPC) image (Fig. 2b), in which both cations and oxygen atoms are directly visible^29^. Combined with atomically resolved energy dispersive X-ray spectroscopy (EDS), which can be used to further check the possible chemical mixing in the GB (Fig. 2c), we ultimately determined the atomic arrangement in the buried GB, as schematically shown in Fig. 2d. Quantitative elemental analysis is also shown in Supplementary Fig. 1 to reveal the local chemistry across the GB, experimentally showing no distinguishable chemical intermixing or segregation. From the iDPC image, the oxygen octahedron distortions are recognizable at a few angstroms wide around the GB. The trapezoid-shaped units in the core structure of the GB impose huge strain gradients and lead to substantial distortion and shifts of AlO columns, intuitively visible to the naked eye.

### Quantitative analysis of nanoscale flexoelectricity

**Fig. 3:**
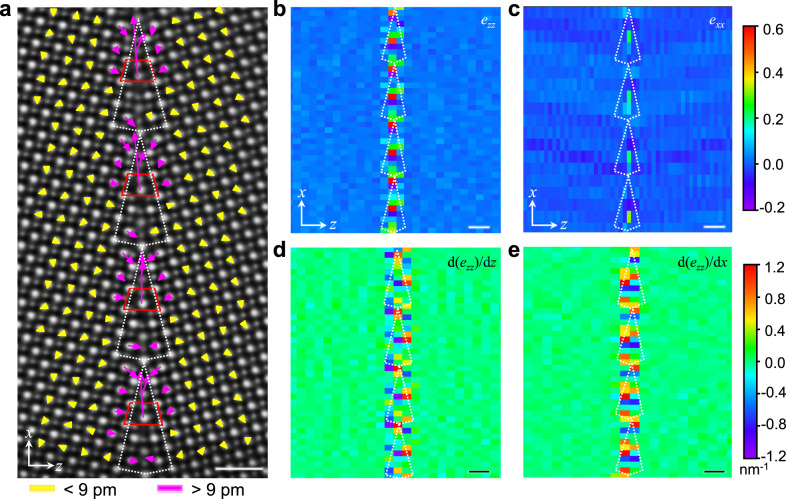
Atomic-scale displacements and strain gradients in the LAO GB. **a** Off-center displacement vector map between the La and AlO columns from the iDPC image. The structural units of the GB are highlighted by the white polygons. The arrows represent the magnitude and direction of polar displacements estimated based on the offsets of the AlO column with respect to the center of the surrounding four La columns. Scale bar, 1 nm. **b**, **c** Unit cell-scale mapping of *e*_*zz*_ (**b**) and *e*_*xx*_ (**c**) corresponding to the strain perpendicular and parallel to the GB plane, respectively. Scale bar, 1 nm. **d**, **e** Strength of the strain gradients from *e*_*zz*_. **d**, **e** Horizontal (d(*e*_*zz*_)/d*z*) and vertical (d(*e*_*zz*_)/d*x*) strain gradients, respectively. The structural units of the GB are highlighted by the white polygons. Scale bar, 1 nm.

**Fig. 4:**
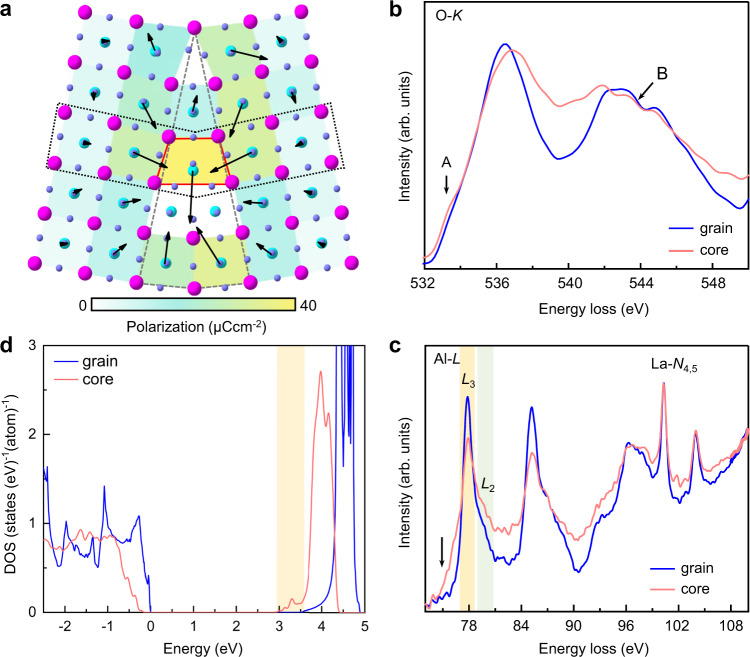
Polarization and electronic structures of the LAO GB. **a** Atomic structure and polarization of the GB from DFT calculations. Vectors denote the polarization direction for each unit cell. The strength of polarization is expressed as a color map, ranging from white (weak) to yellow (strong). In the red polyhedron, the displacement between the cations and oxygen atoms in the AlO octahedron is clearly visible with an upward component, accounting for the deviation between the polar vector and displacement vector. **b** O *K*-edges from the GB core (red) and grains (blue) indicating stronger hybridization of La–O interactions in the GB. **c** Al *L*-edges and La *N*-edges manifesting different local Al–O configurations in the GB. The corresponding Al *L*_3_ (yellow) and *L*_2_ (green)-edges are marked for clarity. **d** Calculated density of states of the GB core (red) and grains (blue). The valence band maximum (VBM) energies in the bulk and at the GB are set to zero.

To better characterize the flexoelectric effect at the GB, a map of atomic displacements was extracted based on the iDPC image, as illustrated in Fig. 3a, wherein the overlaid vectors represent the magnitude and direction of the AlO column offsets with respect to the center of the surrounding four La columns. Such a cationic displacement measurement can minimize the tilt effect^30^, which is inevitable for GB measurement, as the two grains are not exactly identical in terms of zone axes (see details in the Methods and Supplementary Fig. 2). A “head-to-head” configuration pattern of polar vectors emerges around the GB. Note that the magnitude of these vectors is typically higher than the level of noise and possible artifacts (see details in the “Methods” section). To further evaluate the flexoelectric performance of the GB, we quantitatively mapped the strain and strain gradient based on the cation sublattices in the iDPC image (Fig. 3b–e). The surrounding grains away from the GB were regarded as the reference of a perfect (unstrained) crystal. The strength of the strain parallel to the GB plane (*e*_*xx*_) is small (Fig. 3c), while the strain gradients from *e*_*zz*_ along the horizontal (d(*e*_*zz*_)/d*z* in Fig. 3d) and vertical (d(*e*_*zz*_)/d*x* in Fig. 3e) directions are noticeable within 1 unit cell away from the structural unit. Such a highly nonuniform strain distribution around the GB core is also verified by the geometric phase analysis (GPA) in Supplementary Fig. 3^31^. In particular, the unique core structure of the GB (marked by the red trapezoid in Figs. 3a and 4a) undergoes drastic fluctuation of the strain gradient up to 1.2 nm^−1^ (d(*e*_*zz*_)/d*x*), producing a significant polar displacement (~81.4 ± 10.5 pm) which is expected to generate large flexoelectric polarization (more details on error estimation are included in the “Methods” section), while the strain gradients d(*e*_*zz*_)/d*z* are negligible due to the symmetric geometry along this direction. For the shear strain, its contribution to the overall flexoelectricity should be very small according to previous theoretical studies^32^.

Based on the DFT calculation in Fig. 4a, the maximum polarization of this trapezoidal-shaped unit is evaluated as ~38 μC cm^−2^. The longitudinal flexoelectric coefficient *f*_11_ of LAO can then be estimated as 0.3 nC m^−1^, manifesting reasonable agreement with the values in a previous work obtained from theoretical calculations of similar materials^25^. Despite the small difference due to the complex environment of the GB core, the overall atomic structure in the GB presented by DFT calculations is in decent agreement with the experimental results, as illustrated in Supplementary Fig. 4. Hence, the simple consideration of the off-centering between cationic columns delivers the main features of the polar vector distribution, except for the red polyhedron in Figs. 3a and 4a, in which the calculation shows that the displacement between the cations and oxygen atoms in the AlO octahedron has an upward component and dominates the polar vector, causing a discrepancy with the experimental cationic displacement results. Interestingly, the polarizations around the GB also point toward the GB core, forming a “head-to-head” configuration with an average value of ~10 μC cm^−^^2^ from each grain side, analogous to the charged domain wall in ferroelectrics. Furthermore, the charge density distribution calculated by DFT simulations (Supplementary Fig. 5a) shows that electrons have a sharp maximum value at the GB, which can effectively screen the depolarizing fields and stabilize the head-to-head polarization configurations. Moreover, four-dimensional STEM (4D-STEM) measurements (see details in the “Methods” section) in Supplementary Fig. 5b–d further validate the presence of negative charge accumulation at the GB, which certainly plays an important role in determining the electrical activities of the GB in these electroceramics.

### Electrical structure of the GB

Therefore, we performed EELS at the oxygen *K*-edge and the aluminum *L*-edge to investigate the electronic structure of the flexoelectric phase in the GB. For reliability and accuracy of the data, we carefully chose clean and uniform regions to perform EELS measurements, as shown in Supplementary Fig. 6 (see details in the “Methods” section). The spectra from the bulk matrix and the GB core are extracted in Fig. 4b, c. The oxygen *K*-edge spectra (Fig. 4b) illustrate a similar peak at 536.5 eV in the bulk and GB core, which is related to La 5*d*–O 2*p* hybridized states^33^, while a feature at approximately 533.5 eV (peak A) only exists in the GB, indicating stronger La–O interactions^34^. Spectral region B corresponds to the transition to O 2*p* states hybridized with Al 3*p* states^34,35^. The different fine structure of spectral region B in the GB demonstrates the modified hybridization between Al and O states. A previous study reported that the hybridization between Ti 3*d* and O 2*p* in PbTiO_3_ and BaTiO_3_ accounts for their ferroelectricity^36^. Therefore, the stronger La–O hybridization in the GB core is in well agreement with the presence of flexoelectricity. Additionally, the Al *L*-edge spectrum generally exhibits a fingerprint that signifies the change in the Al coordination symmetry and reflects the unoccupied conduction band minimum (CBM)^37–39^. As shown in Fig. 4c, the decrease in the threshold energy at the Al *L*_3_-edge highlighted by the arrow is ~0.6 eV, illustrating distorted octahedral symmetry in the GB area^40^, which is also reflected in the difference in the fine structure at the Al *L*_1_-edge (Supplementary Fig. 7a)^41^ and the substantial reduction in the *L*_3_/*L*_2_ ratio (depicted in Supplementary Fig. 7b). Furthermore, because the Al *L*_2,3_-edges originate from the electron excitations from the Al 2*p* to unoccupied Al 3*s* states, while the Al 3*s* orbital dominates the CBM^39^, the lower onset energy may also manifest a band gap reduction in the GB, which is confirmed by DFT calculations of the density of states (Fig. 4d) and electronic band structure (Supplementary Fig. 8).

## Discussion

To validate the generality of the flexoelectricity in the GB, we then investigated SrTiO_3_ (STO) GBs to present the universal structural information associated with adjustable flexoelectricity. As shown in Supplementary Fig. 9, the maximum strain gradient in the 22.6° STO GB is evaluated as ~1.5 nm^−^^1^ for d(*e*_*zz*_)/d*x*, resulting in a cationic atomic shift of up to 60.3 ± 5.7 pm. The strain gradients are more pronounced in the 36.8° STO GB core (~1.9 nm^−^^1^ for d(*e*_*zz*_)/d*x*), inducing giant atomic shifts up to ~119.1 ± 4.3 pm (Supplementary Fig. 10). Note that such an enhancement of displacements might not only be caused by flexoelectricity but also partly come from the nonstoichiometry which are usually strongly coupled with each other^23^. We also measured the EELS spectra for different STO GBs, and found them very different. For example, the 22.6° STO GB shows more decreased crystal field splitting (Supplementary Fig. 11). However, it should also be noted that in addition to the flexoelectricity, the electrical changes in the GB are probably influenced by multiple factors, such as bonding changes, possible local charge changes, and their interactions, rather than only flexoelectricity. On the other hand, the strain gradients in the LAO and STO GB cores measured by experiments (depicted in Fig. 1b and highlighted by red dots), show good consistency with the estimated geometric relation, indicating that a wide range of strain gradients can be obtained by controlling the GB angle. In addition to tunability and universality, GB engineering has advantages in generating flexoelectricity compared with other strategies, such as changing the epitaxial conditions or other defects. For example, although great tensile strains exist in the center of cracks in STO^42^, symmetric stretching prevents the flexoelectric polarization response due to the small strain gradients. In contrast, the GB restricts the large strain gradients to within a few unit cells, serving to deterministically trigger nanoscale flexoelectricity.

Moreover, the polarized GB core further influences the electrostatic potential and the electronic density in the GB, modulating the electrical activities. Taking LAO as an example, a stronger hybridization state is located in the GB core, favoring and promoting the formation of polar distortion. The decrease in the Al *L*_3_ onset energy indicates disordered configuration complexity, which may allow weak *p*–*d* hybridized states that are forbidden in the perfect AlO_6_ octahedral configuration, possibly causing a small number of defective states between the valence band maximum (VBM) and CBM^39,40^. In addition, around the GB, the flexoelectric distortion may also couple with the rotational distortion (e.g., antiferrodistortive distortion at the 22.6° STO GB^21^), giving rise to additional effects such as improper ferroelectricity^43^ and the rotostriction effect^44^ and thus influencing the electronic properties of the GB in a more complicated fashion.

Although GBs is well-known to play critical roles in the properties of oxide ceramics, the underlying mechanism is far from clear due to the intricacy. Among all the proposed mechanisms, elemental segregation-induced local nonstoichiometry^45,46^ is usually believed to be the dominant mechanism, which changes the local charge conditions and influences the transport properties^47–49^. In our study, the observed universal nanoscale flexoelectricity at GBs in these oxide ceramics provides a scenario to understand the electrical activities, i.e., the flexoelectric dipole carries “internal” polarization, and then, the bound charge must be screened by the “external” charges or it will not stably exist^50^, inducing charge (electron, oxygen vacancy, hole, etc.) redistribution and thus being expected to likewise influence the electrical activities. Furthermore, we built a structure that only has off-stoichiometry without huge strain gradients, showing a much smaller dipole moment (~9 μC cm^−^^2^) compared with the polarization of the trapezoidal cell in the GB core. Hence, the unique trapezoidal unit cell-induced flexoelectricity has a significant influence on the polar displacements and hence electrical activity. In this sense, the role played by the huge strain gradient-induced flexoelectricity is as important as the commonly believed elemental segregation for GBs.

In summary, we establish a systematic picture of nanoscale flexoelectricity at GBs, in which tunable and huge strain gradients can be achieved at the atomic scale. By the advanced STEM technique and theoretical DFT calculations, we determine the overall atomic structure of the exotic trapezoidal-like configuration in the GB, evaluate the strain gradients, quantify the atomic-scale flexoelectricity, reveal the electronic structure, and demonstrate the generality. Due to the huge strain gradients, large off-displacements of more than a hundred picometers can be achieved in the GB core with a polarized area typically within 3~4-unit cells. The presence of considerable flexoelectricity in the GBs and its universality provide insights into understanding the electrical activities of GBs in electroceramics.

## Methods

### Fabrication of bicrystals

A LAO bicrystal with a 24° mistilt GB was fabricated by using thermal diffusion bonding of two LAO single crystals at 1800 °C for 12 h in a N_2_ atmosphere under a uniaxial stress of 2 MPa. A STO bicrystal with a 36° mistilt GB was fabricated based on thermal diffusion bonding of two STO single crystals at 1000 °C for 10 h in a N_2_ atmosphere under a uniaxial stress of 2 MPa. The bicrystal was supplied by Hefei Ke Jing Materials Technology Co., LTD.

### Preparation of TEM samples

The TEM specimens were first thinned by mechanical polishing and then subjected to argon ion milling. The ion milling process was carried out using a PIPS™ (Model 691, Gatan, Inc.) with an accelerating voltage of 3.5 kV until a hole was observed. Low voltage milling was performed with an accelerating voltage of 0.3 kV to remove the surface amorphous layer and minimize the irradiation-damaged layers.

### Electron microscopy characterization and image analysis

HAADF and iDPC images were recorded at 300 kV using an aberration-corrected FEI Titan Themis G2. The convergence semiangle for imaging was 30 mrad, and the collection semiangle snap range was 4–21 mrad for iDPC imaging and 39–200 mrad for HAADF imaging. To obtain a sufficient signal-to-noise ratio for quantitative analysis, the iDPC image was acquired at 4096 × 4096 pixels, with a dwell time of 500 ms pixel^−1^ and a beam current of 20 pA to avoid beam damage.

To obtain quantitative information on the flexoelectricity, the atom positions were determined by simultaneous fitting with two-dimensional Gaussian peaks using a MATLAB code. The magnitude of the strain was defined as the difference in the lattice parameters between the strained and ground states. The strain was calculated based on the La (Sr) sublattice as ${{{{{{\mathrm{strain}}}}}}}=\frac{a\,-\,{a}_{0}}{{a}_{0}},$ where *a*_0_ is the lattice parameter of free-standing LAO (STO) and *a* is the in-plane lattice parameter perpendicular (*a*_*z*_) or parallel (*a*_*x*_) to the GB plane. The strain gradient was then obtained by taking the differential of the strains along different directions, given as d(*e*_*zz*_)/d*z* = 2 * (${e}_{{zz}}^{n}$ − ${e}_{{zz}}^{n-1}$)/(${a}_{z}^{n}$ + ${a}_{z}^{n-1}$) and d(*e*_*zz*_)/d*x* = (${e}_{{zz}}^{m}$ − ${e}_{{zz}}^{m-1}$)/${a}_{x}^{m}$). In Fig. 1b, we recognize side *c* as the lattice parameter (*a*_0_) of the free-standing state. GPA was performed using the free FRWRtools plugin for Digital Micrograph (developed by Kotch et al. and available at https://www.physics.hu-berlin.de/en/sem) based on the original work^31^.

A previous study^51^ suggested that the error in displacement measurement mainly comes from misalignments (e.g., specimen mistilt and residual lens aberration). In our work, we conducted careful alignment to minimize the artifacts. We set the center of the Ronchigram of the GB to the alignment center when acquiring the image, as shown in Supplementary Fig. 2a. The corresponding Ronchigrams of two surrounding grains are also shown in Supplementary Fig. 2b, c. Then, the offset between the center of the Ronchigram of surrounding grains and the alignment center indicates the deviation from their on-zone axis orientations (nearly 1.7 mrad, corresponding to 0.1°). Based on the experimental parameters, we performed a QSTEM software simulation (https://www.physics.hu-berlin.de/en/sem/software/software_qstem). The results show that such crystal tilts will result in ~1 pm false displacement measurements (Supplementary Fig. 2d). Furthermore, the surrounding grains away from the GB core were regarded as the reference polarization-free structure and analyzed to evaluate the mistilt effect. Then, the displacements in the reference LAO and STO were measured as 4.3 ± 1.8 pm (Supplementary Fig. 2e) and 4.8 ± 1.6 pm, showing good agreement with the previous iDPC image error level (typically ~5 pm^51^). The discrepancy between the simulation and experiment mainly arises because the experimental error comes from not only mistilt noise but also scanning noise and specimen drift in acquiring STEM images^30^. Nevertheless, the error level is typically one order below our flexoelectric displacement (~81.4 pm), proving the data reliability. To further minimize the fluctuation between different locations, the estimated displacements in the GB core were averaged among the periodic structural units to present the key features.

### Four-dimensional scanning electron diffraction (4D-STEM) characterizations

Scanning diffraction data sets were collected by using a Nion HERMES 200 microscope operated at 60 kV. The probe convergence semiangle was 35 mrad. The sample thickness was estimated as ~6 nm. All acquired 4D data sets were processed by custom-written Python code based on the GetDPC package^52^. However, such phase contrast in 4D-STEM mode is very sensitive to the crystal tilt^53^ and thickness variation and limit^54^. Thus, achieving an accurate conclusion regarding the quantitative charge analysis for GBs from 4D-STEM measurements remains challenging due to the presence of small misalignment between two grains, stress relaxation in the ultrathin specimen, and possible thickness differences across the GB.

### EELS characterizations and analysis

STEM-EELS spectra were recorded using a Nion HERMES 200 microscope. To reduce beam damage, EELS experiments were performed at 60 kV. The probe convergence semiangle was 35 mrad, and the collection semiangle was in the range of 24.9 mrad. We acquired a single 25 × 70 pixel EELS map from a 4.27 × 16 nm region containing the GB. The dwell time was 150 ms pixel^−1^, and the dispersion was 0.1663 eV ch^−1^. The data were smoothed by a Gaussian-weighted moving average filter with a window length of 7 channels and a variance of 1 channel. The extracted EELS spectra in Fig. 4b, d were spatially averaged over the regions parallel to the GB plane (marked by the rectangle in Supplementary Fig. 6b). The EELS background was fitted and subtracted using the power law $I\left(\triangle E\right)={A}_{0}\cdot {\triangle E}^{-r}$. The *L*_3_/*L*_2_ ratio calculation was based on the spectral integral intensity ratio of the 77.2–78.8 eV and 79.3–80.9 eV energy windows. The fitting uncertainty was estimated by the standard deviation among rows.

### First-principles calculations

Electronic structure calculations were performed using the Vienna Ab initio Simulation Package (VASP)^55,56^. The projected augmented wave (PAW)^57,58^ potential was used to address with the interactions between ions and electrons. The exchange-correlation potential was treated by the Perdew–Burke–Ernzerhof^59^ functional. A plane-wave cutoff of 600 eV was used. For the bulk rhombohedral structure, the experimental lattice constants (*a* = *b* = *c* = 5.360 Å, *α* = *β* = *γ* = 60.011°) were used. Then, the bulk pseudocubic structure was used as a block unit to build the GB structure^60^. Atomic positions were relaxed until the maximum force was less than 0.01 eV Å^−1^. Band structures were obtained at the DFT level. For the GB, we found that the charge-neutral system is gapless. Thus, to make the system have a finite gap, extra four electrons were added to the cell. Born effective charges were computed using density functional perturbation theory^61–63^.

The polarization calculation was based on the equation of spontaneous polarization *P*_*s*_ = $\frac{1}{V}\sum {\delta }_{i}{Z}_{i}$^64^, where ${V}$ is the volume of the unit cell, $\delta$ is the displacement of atom, $i$ from its centrosymmetric position and $Z$ is the Born effective charge of atom $i$. $\delta$ was evaluated from the displacements of each column with respect to the corresponding position in the polarization-free bulk crystal. The charge density was calculated within the DFT in the VASP package.

## Supplementary information

Supplementary Information

## Data Availability

The authors declare that all relevant data are included in the paper and Supplementary Information files and are available from the corresponding author upon reasonable request.

## Code availability

Additional data including the codes are available from the corresponding author upon reasonable request.
