# Supplementary material for: Engineering of atomic-scale flexoelectricity at grain boundaries
Source: Nat Commun. 2022 Jan 11;13:216. doi: 10.1038/s41467-021-27906-0 (PMC8752668; doi:10.1038/s41467-021-27906-0)
Supplement: Supplementary file 1 — Supplementary Information [file 41467_2021_27906_MOESM1_ESM.pdf]

Supplementary Information for

Engineering of Atomic-Scale Flexoelectricity at Grain Boundaries

Mei Wu[1,2†], Xiaowei Zhang[1†], Xiaomei Li[1,3], Ke Qu[2], Yuanwei Sun[1,2], Bo Han[1,2], Ruixue Zhu[1,2], Xiaoyue Gao[1,2], Jingmin Zhang[2], Kaihui Liu[4,5,6], Xuedong Bai[3], Xin-Zheng Li[4,5,7,8*] and Peng Gao[1,2,5,6*]

Correspondence should be addressed to
Email: p-gao@pku.edu.cn (P.G.); xzli@pku.edu.cn (X.Z.L.)

This PDF file includes:

Supplementary Figs. 1 to 11

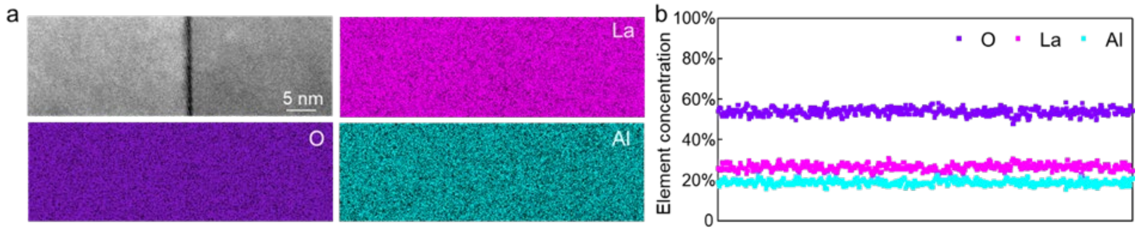

Supplementary Fig. 1 Quantitative elemental analysis of the GB. The sample was tilted by 15° to reduce complex electron scattering effect while maintaining edge-on conditions for the GB[1]. **a** The acquired HAADF image and EDS concentration maps (atomic %) for O (purple), La (magenta) and Al (cyan). **b** The corresponding intensity profiles of EDS concentration (atomic %) for O (purple), La (magenta) and Al (cyan) across the GB. It is clear that for O, Al and La, there are not any distinguished concentration changes within the noise range across the GB.

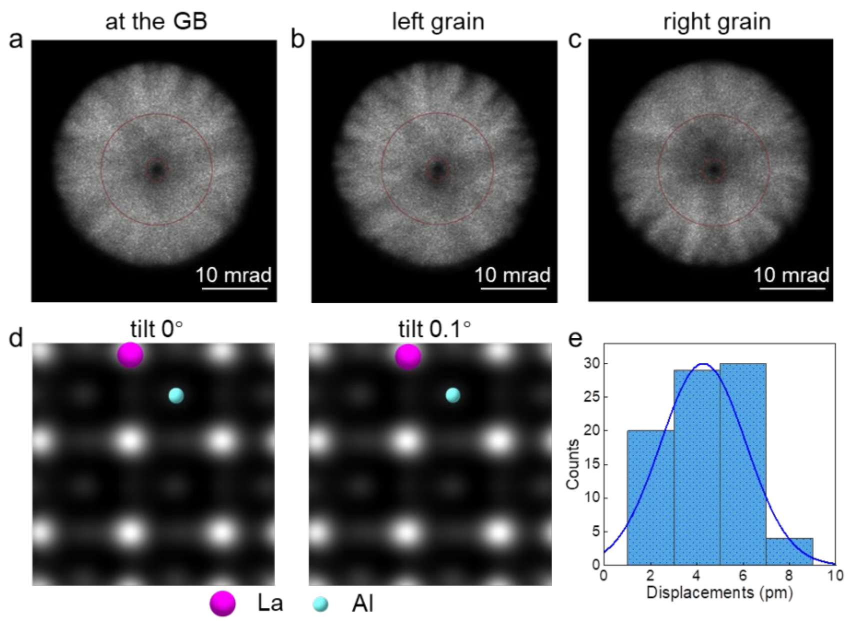

Supplementary Fig. 2 a,**b**,**c** The corresponding Ronchigrams at the GB (**a**), the left grain (**b**) and right grain (**c**). **d** Simulated HAADF images by QSTEM software [2] along [001] direction at tilt 0° (left panel) and 0.1° (right panel) based on the experimental parameters. **e** Cationic displacements between AlO column with respect to the center of their surrounding four La columns for the surrounding grains away from the GB core based on the iDPC image of Fig. 3a.

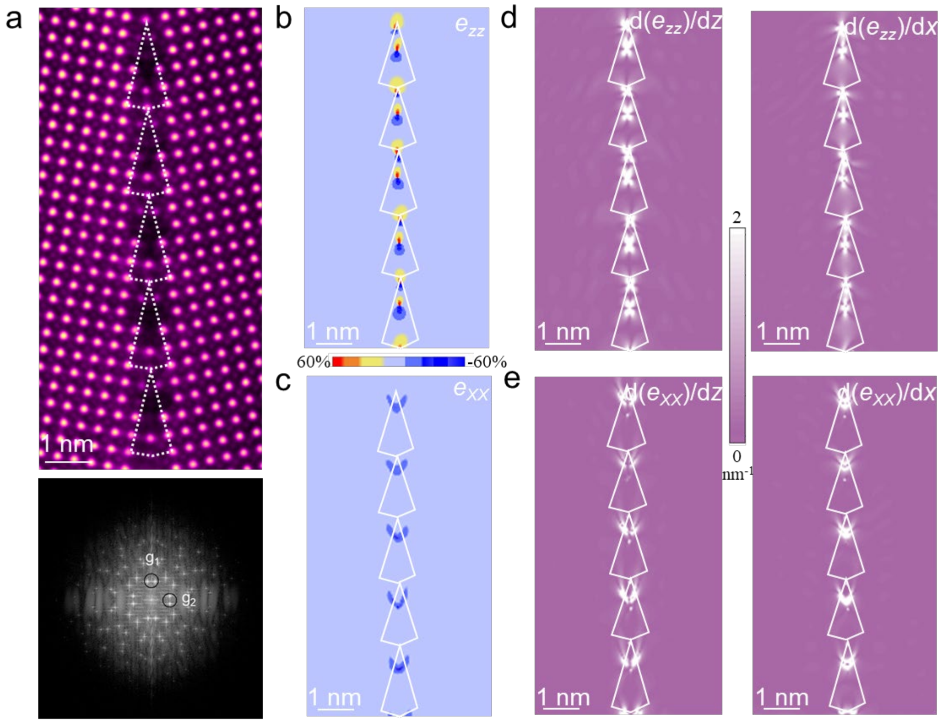

Supplementary Fig. 3 GPA of the GB. a HAADF image. The corresponding GPA parameter are shown in the bottom panel. The two black circles represent the reflection g_1 and g_2. The resolution is ~ 1 nm. **b,c** GPA showing the distribution of the strain (**b**) e_{zz} perpendicular to the GB plane and (**c**) e_{xx} parallel to the GB plane. **d,e** Calculated strength of strain gradients from (**d**) e_{zz} and (**e**) e_{xx}. Left and right panel corresponds to the horizontal and vertical strain gradients respectively in (**d**) and (**e**).

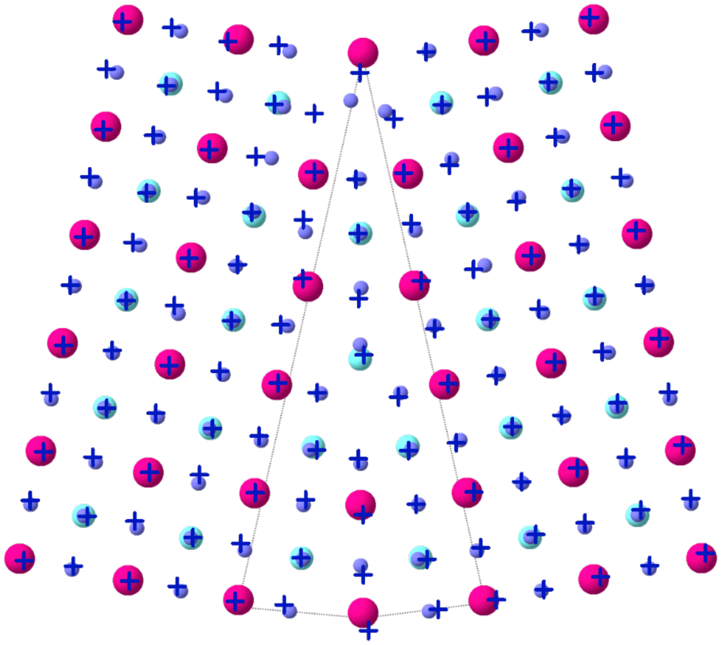

Supplementary Fig. 4 Comparison between experiments and DFT model of the atomic arrangements for GB. The cross represents the averaged spatial distributions of the four structural units in Fig. 3a. The atomic structure is based on the DFT calculations, containing La (magenta), Al (cyan) and O (purple) atoms.

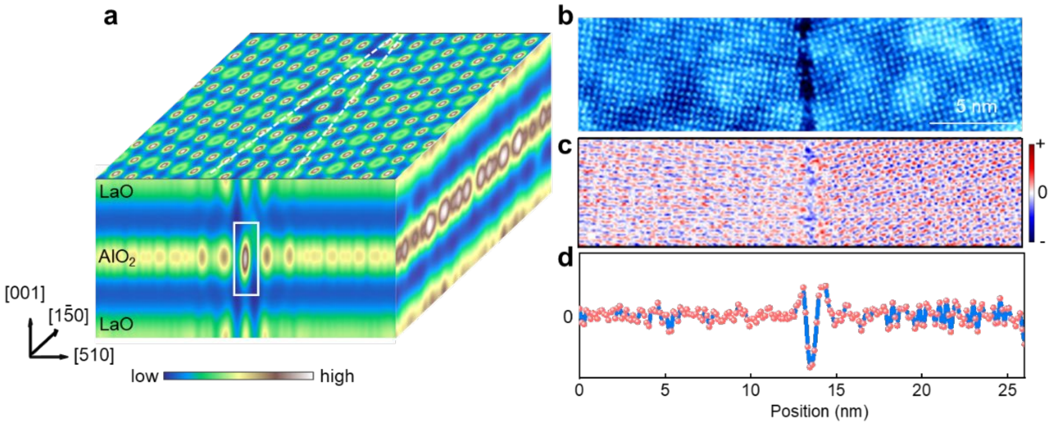

Supplementary Fig. 5 The charge density distribution around the GB. a The calculated electron charge density distribution by the DFT in the VASP package. As marked by the white rectangle, electrons have a sharp maximum value at the GB. **b** Simultaneous recorded HAADF image in 4D STEM acquirement. **c,d** The charge density mapping (**c**) measured by 4D STEM and corresponding line profiles of charge variations (**d**) integrating the measured charge density vertically. We choose the clean and thin region containing the GB to perform the 4D STEM measurements. The crystal tilt between two grains is nearly 1.7 mrad. The thickness of the specimen in the regions was evaluated to be ~ 6 nm based on log-ratio methods from the low-loss EELS of corresponding sample regions. The error bar is the standard derivation.

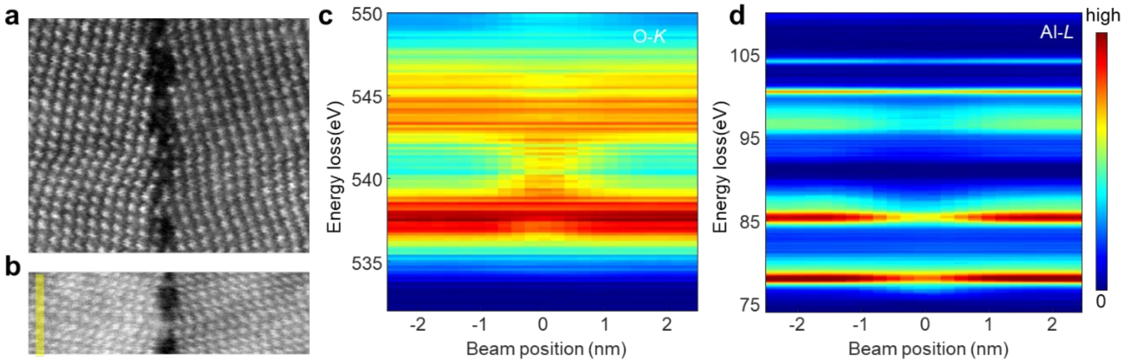

Supplementary Fig. 6 a The acquired HAADF image of GB before EELS acquirements. **b** the HAADF image after EELS acquirements, showing no obvious beam damage. **c,d** The extracted EELS line profiles of O-*K* (**c**) edges and Al-*L* (**d**) edges across the GB, which are spatially averaged over the regions indicated by the yellow rectangle in (**b**).

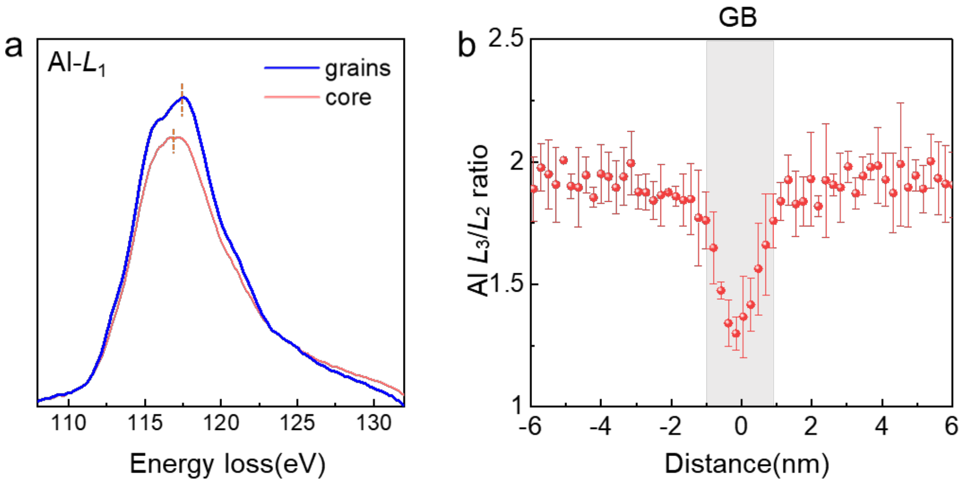

Supplementary Fig. 7 Electronic structures of the LAO GB. a Al-L_1 edges indicating different local Al-O topological configuration in the GB. **b** The change of Al $L_{2,3}$ ratio across the GB, indicating the spatial scale of the octahedral distortions and changed local Al-O environments is ~ 2 nm. The error bar is the standard derivation.

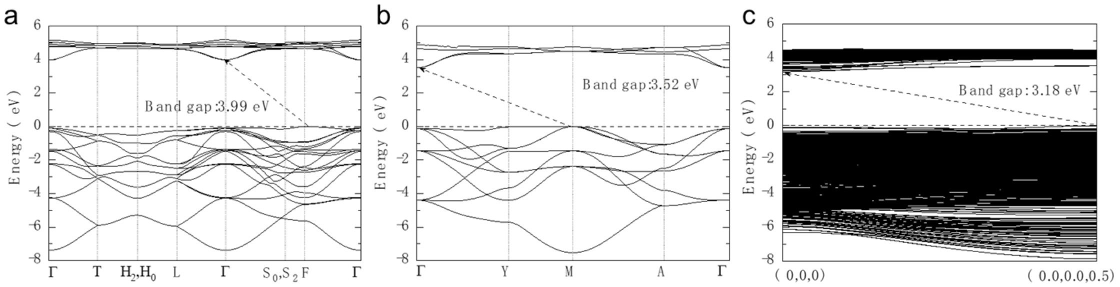

Supplementary Fig. 8 Theoretically calculated band structures of LAO for bulk phase and GB. a bulk rhombohedral structure. **b** similar to (**a**) but adopted the pseudo-cubic unit cell. **c** GB structure. In (**a**) the band gap is indirect, which is different from the previously reported result. We attribute this to slightly different atomic positions and lattice constants in different calculations [3]. The DFT band gaps are greatly underestimated due to the use of semi-local functional. But, fortunately, we are concerned about the band gap change from bulk to GB and we expect that more accurate electronic structure methods should not have large corrections to this change. Since the GB is built up from the pseudo-cubic unit cell, the band gap change should be obtained between (**b**) and (**c**), i.e. 0.34 eV.

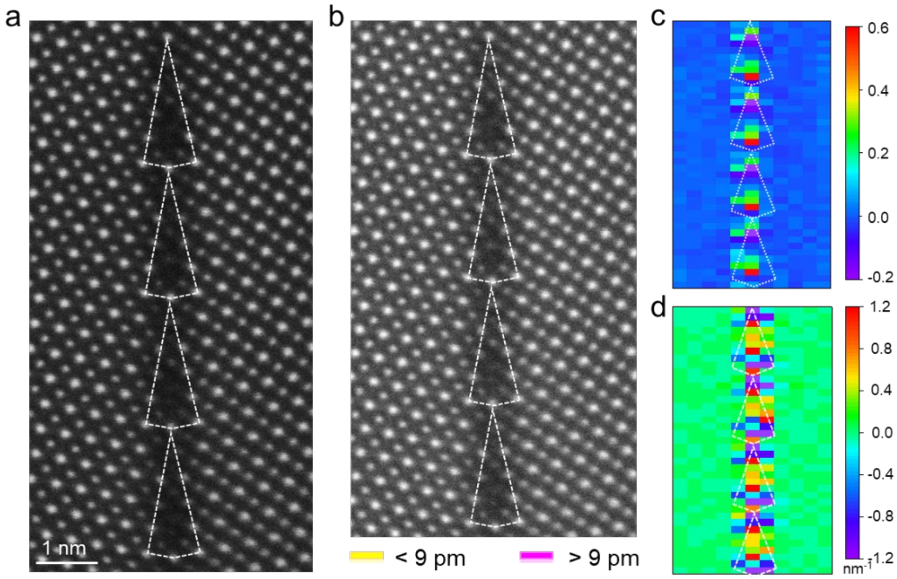

Supplementary Fig. 9 Polarization in the 22.6° STO GB. a A HAADF image of STO 22.6° tilt GB. The polygon highlights the structure unit of GB. **b** Displacement vector map between the Sr and Ti columns based on the HAADF image. **c** The unit cell scale mapping of e_{zz} corresponding to strain perpendicular to the GB plane. **d** Strength of strain gradients from e_{zz} in the vertical direction [d(e_{zz})/dx]. The structural units of the GB are highlighted by the white polygon.

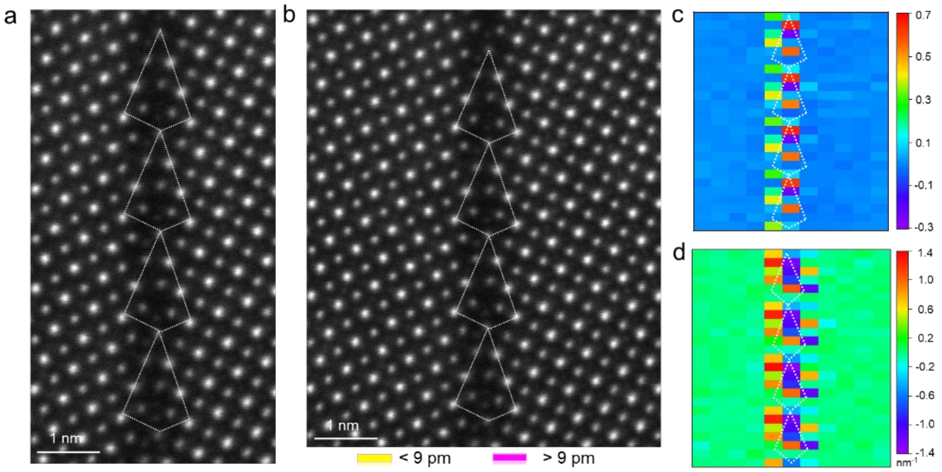

Supplementary Fig. 10 Polarization in the 36.8° STO GB. a A HAADF image of STO 36.8° tilt GB. The polygon highlights the structure unit of GB. **b** Displacement vector map between the Sr and Ti columns based on the HAADF image. **c** The unit cell scale mapping of e_{zz} corresponding to strain perpendicular to the GB plane. **d** Strength of strain gradients from e_{zz} in the vertical direction [d(e_{zz})/dx]. The structural units of the GB are highlighted by the white polygon.

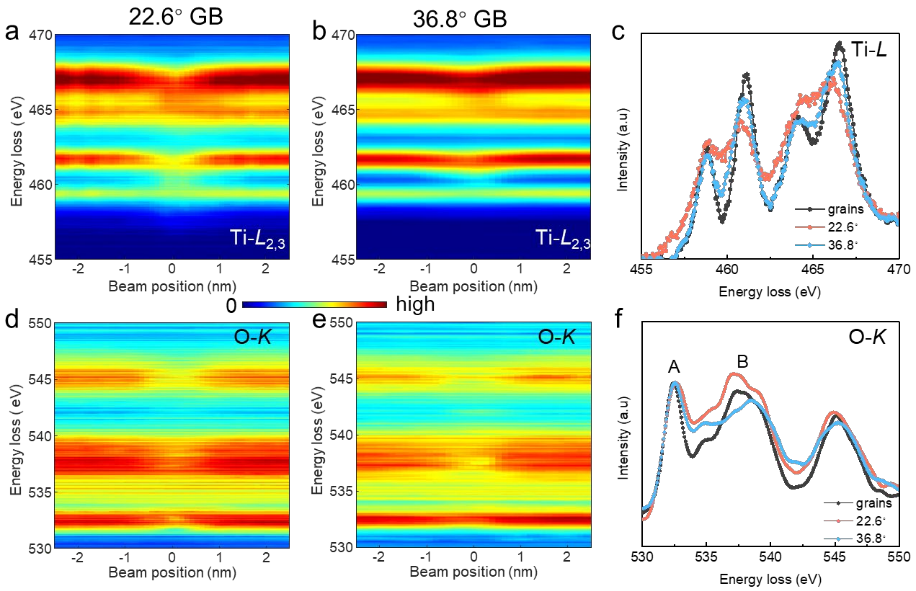

Supplementary Fig. 11 Electronic structure of the STO GBs. a,**b** Ti-$L_{2,3}$ edge across the 22.6° (**a**) and 36.8° (**b**) STO GB. **c** The extracted Ti-$L_{2,3}$ edges at STO grains (black), 22.6° (red) and 36.8° (blue) STO GB. The 22.6° STO GB shows more decrease of crystal field splitting feature. **d**,**e** O-K edge across the 22.6° (**d**) and 36.8° (**e**) STO GBs. **f** A comparison of O-K edge at STO grains (black), 22.6° (red) and 36.8° (blue) STO GB. Peak A becomes more broaden and the fine structure of peak B shows more changes at the 22.6° STO GB.

References

1. Feng, B., Lugg, N. R., Kumamoto, A., Ikuhara, Y. & Shibata, N. Direct Observation of Oxygen Vacancy Distribution across Yttria-Stabilized Zirconia Grain Boundaries. ACS Nano 11, 11376-11382 (2017).
2. The QSTEM simulation is based on Christoph Koc's dissertation, "DETERMINATION OF CORE STRUCTURE PERIODICITY AND POINT DEFECT DENSITY ALONG DISLOCATIONS." See also https://www.physics.hu-berlin.de/en/sem/software/software_qstem. and https://www.physics.hu-berlin.de/en/sem/images/koch02_phdthesis.pdf.
3. X. Luo, B. Wang, First-principles study of the electronic and optical properties in rhombohedral $LaAlO_3$. *J. Appl. Phys.* **104**, (2008).
